# Supplementary material for: The Effects of Risk Perceptions Related to Particulate Matter on Outdoor Activity Satisfaction in South Korea
Source: Int J Environ Res Public Health. 2020 Mar 2;17(5):1613. doi: 10.3390/ijerph17051613 (PMC7084646; doi:10.3390/ijerph17051613)
Supplement: Supplementary file 1 [file ijerph-17-01613-s001.pdf]

Supplementary Material.

This survey system created a survey site[1] using a survey form provided by Google. The survey site was linked to five Internet communities, who agreed to participate after revealing the purpose of this study. To prevent duplicate responses, individual phone numbers were collected after the respondents agreed. When the questionnaire was finished, the responses were stored in a database through Google Drive, and the survey results were converted into data that could be analysed using the statistical package.

The survey consisted of a pilot test and main survey. Three pilot tests were conducted in June 2019 by approximately 15 researchers with master's and doctoral degrees. After collecting and supplementing the questionnaire's problems and system malfunctions to improve the response rate and completeness, the main survey was completed.

Data collection for the main survey was conducted for about one month from July 16 to August 16, 2019. During this period, the concentration level of PM in Korea was low and the posts related to PM were only a few. The reason for choosing this period was that we wanted to ensure that the experienced PM risk perception was not greatly influenced by the PM concentration in the surrounding environment. The survey was written in the form of a single page to increase response rates and completeness and re-submitted survey posts to the Internet communities every 10 days. In addition, sub-items were organized by dividing sections into topics for the recognition of items and ease of answers. In the case of non-response items, the survey was not terminated so that missing values could be minimized. In other words, if the respondents did not complete the survey, the questionnaire was not stored in Google Drive. Finally, a total of 412 copies were collected.

The questionnaire consisted of five parts: basic knowledge about PM, characteristics of outdoor activities related to PM, PM risk perception, outdoor activity satisfaction, and respondents' descriptive statistics. In order to understand the sample characteristics, the respondents' descriptive statistical questionnaires, such as gender, age, and residence, consisted of a categorical nominal scale and frequency analysis was conducted. The 5-point Likert Scale was used for the PM risk perception and outdoor activity satisfaction. In the analysis process, Microsoft Office Excel 2017 and SPSS 21.0 (SPSS Inc., 2013) were used.

Table 1. Questionnaire.

Cognitive questions
Section 1. Basic knowledge about PM<br>This is a question to find out the Basic knowledge about PM of survey respondents. PM in the lower question is a generic term for fine dust (PM_{10}) and ultra-fine dust ($PM_{2.5}$). Please respond to all items. (Approx. 2 minutes, 4 questions)<br>1. Do you know what PM is?<br>2. Do you know the difference between fine dust (PM_{10}) and ultra-fine dust ($PM_{2.5}$)?<br>3. When you started to recognize PM?<br>4. How do you usually get PM information? (Multiple)
Section 2. Outdoor activities related to PM<br>PM in the lower question is a generic term for fine dust (PM_{10}) and ultra-fine dust ($PM_{2.5}$).<br>Please answer all questions. (Approx. 2 minutes, 5 questions)

[1] Internet survey address https://forms.gle/RU6hBWrV9WUmcunYA. The site has not been operated since the survey period was over.

1. What are your regular outdoor activities?
2. Is there a PM concentration standard when planning outdoor activities?
3. What health discomforts are mainly caused by PM? (Multiple)
4. What are the countermeasures for outdoor activities under high PM concentration?
5. How can you deal with outdoor activities when high concentration levels of PM occur?

Section 3. PM risk perception

This is a question about the cause of risk perception in outdoor activities caused by PM.
Please respond to all items. (Approx. 5 minutes, 12 questions)

1. To what extent do you think about experienced perception changes regarding PM concentration during OA?
2. To what extent do you trust the PM concentration provided by the government?
3. To what extent do you think about health risks of experienced fine dust (PM_{10})?
4. To what extent do you think about health risks of experienced ultra-fine dust ($PM_{2.5}$)?
5. To what extent do you spend on average to find PM information?
6. To what extent do you trust the PM information provided by the public opinion?
7. To what extent do you interest in public opinion related to PM?
8. To what extent do you interest in the PM policies implemented by the government?
9. [Before 2014_before the national alert system text message sent]
   To what extent do you interest in the public opinion and policies?
10. [After 2014_after the national alert system text message sent]
   To what extent do you interest in the public opinion and policies?
11. To what extent do you trust the domestic PM policies implemented by the government?
12. To what extent do you trust the international PM policies implemented by the government?

Section 4. Outdoor activity satisfaction

Outdoor activities in this research are mainly recreational or physical activities in natural spaces such as mountains, riversides, and parks. It also includes recreational or physical activity in outdoor mobility spaces such as bicycle roads, promenades.

PM Concentration is "unhealthy" or higher (very unhealthy) means more than fine dust (PM_{10}) 81μg / ㎥ or more than ultra-fine dust ($PM_{2.5}$) 35μg / ㎥ according to Korea's fine dust forecasting and alarming system (FDFAS). Please respond to all items. (Approx. 5 minutes, 16 questions)

1. [Although PM concentration is more than unhealthy] My OA helps me to relax.
2. [Although PM concentration is more than unhealthy] My OA helps to relieve stress.
3. [Although PM concentration is more than unhealthy] My OA is good for emotional well-being.
4. [Although PM concentration is more than unhealthy] I participate because of the outdoor fun.
5. [Although PM concentration is more than unhealthy] My OA promotes social exchange.
6. [Although PM concentration is more than unhealthy] My OA develops intimate relationships with others.
7. [Although PM concentration is more than unhealthy] The people I met through my OA are friendly.
8. [Although PM concentration is more than unhealthy] I hang out with people who enjoy their free time.
9. [Although PM concentration is more than unhealthy] My OA is a physical challenge.
10. [Although PM concentration is more than unhealthy] My OA improves my fitness.
11. [Although PM concentration is more than unhealthy] My OA helps me to recover physically.
12. [Although PM concentration is more than unhealthy] MY OA helps me to stay healthy.
13. [Although PM concentration is more than unhealthy] My OA place is comfortable.
14. [Although PM concentration is more than unhealthy] My OA place is interesting.
15. [Although PM concentration is more than unhealthy] My OA place is beautiful.
16. [Although PM concentration is more than unhealthy] My OA place is well designed.

Section 5. Respondents' descriptive statistics

We want to investigate basic information for respondents' descriptive statistics. Responses to this survey will be treated anonymously and treated as statistical figures for purely research purposes only and will not be used for any other purpose. Please respond to all items. (Approx. 2 minutes, 5 questions)

1. Where do you live?
2. What is your gender?
3. What is your age range?
4. What is your marital status?
5. Are there any members of your family who are vulnerable to PM? (Multiple)

Please leave your mobile number. ex) 010-1234-5678

All surveys have been completed. Press Submit to complete the response.
